# Differential Expression Patterns of EGF, EGFR, and ERBB4 in Nasal Polyp Epithelium

**DOI:** 10.1371/journal.pone.0156949

**Published:** 2016-06-10

**Authors:** Chen Duan, Chun Wei Li, Li Zhao, Somasundaram Subramaniam, Xue Min Yu, Ying Ying Li, De Hua Chen, Tian Ying Li, Liang Shen, Li Shi, De Yun Wang

**Affiliations:** 1 Department of Otolaryngology, The Second Hospital of Shandong University, Jinan, China; 2 Department of Otolaryngology, National University of Singapore, National University Health System, Singapore, Singapore; 3 Department of Otorhinolaryngology, The First Affiliated Hospital of Sun Yat-Sen University, Guangzhou, China; 4 Department of Otolaryngology, Ng Teng Fong General Hospital, Singapore, Singapore; 5 Department of Otolaryngology, Qilu Hospital, Shandong University, Jinan, China; 6 Biostatistics Unit, National University of Singapore, National University Health System, Singapore, Singapore; The Perinatal Institute, Cincinnati Children's Hospital Medical Center and University of Cincinnati, UNITED STATES

## Abstract

Epidermal growth factor receptors play an important role in airway epithelial cell growth and differentiation. The current study investigates the expression profiles of EGF, EGFR and ERBB4 in patients with nasal polyps (NP), and their response to glucocorticosteroid (GC) treatment. Fifty patients with NP (40 without GC treatment and 10 with oral GC) and 20 control subjects with septal deviation were recruited into the study. Protein levels of EGF, EGFR, and ERBB4 were evaluated by immune-staining. In healthy nasal epithelium, EGF and EGFR localized within p63^+^ basal cells, while ERBB4 localized within ciliated cells. GC-naïve NP epithelium showed weak expression of EGF in 90% of samples versus 5% of controls. EGFR was significantly increased in the epithelium with basal cell hyperplasia from GC-naïve NPs (78%, 31/40) compared to controls (23%, 4/17). EGFR was also found in some degranulating goblet cells. ERBB4 expression was significantly higher in hyperplastic epithelium from GC-naïve NPs (65%, 26/40) than in controls (6%, 1/17). GC treatment restored the EGF expression and normalized the EGFR and ERBB4 expression in NPs. Differential expression patterns of EGF, EGFR, and ERBB4 are essential in epithelial restitution and remodeling in nasal epithelium.

## Introduction

Human nasal epithelium is the first line of defence against environmental agents, which include viruses, bacteria, allergens and toxic irritants. In response to these damaging agents, the nasal epithelium constantly repairs itself in order to maintain its integrity. The nasal epithelium is composed of three cell types: basal cells, goblet cells, and ciliated or non-ciliated columnar cells. In response to injury, basal cells can proliferate and migrate to damaged sites, and subsequently differentiate to restore all cell types. Therefore, basal cells are considered to be the adult stem cells in the airway, and play a critical role in epithelial repair. However, chronic inflammation of the nasal mucosa results in epithelial repair dysregulation. Nasal polyps (NPs) are characterized by mucosal inflammation, high concentrations of inflammatory cells, and aberrant epithelial remodeling. The NP epithelium is constantly attacked by inflammatory mediators, leading to epithelial damage. Subsequently, the epithelium becomes susceptible to environmental agents, resulting in defective epithelial repair. In addition, pro-inflammatory signals may lead to uncontrolled remodeling of NP epithelium. Our previous studies have demonstrated that *activation protein 1* (AP-1) and its related genes were downregulated in NPs and associated with epithelial damage [1], while p63 was upregulated in epithelial hyperplasia [2]. Therefore, rapid repair of damaged epithelium without causing uncontrolled re-epithelialization is critical to the resolution of inflammation in NP.

The epidermal growth factor receptor signaling system plays an important role in epithelial cell growth, proliferation, and differentiation [3–5]. This network includes a family of growth factors (ligands) and the related tyrosine kinase receptors ERBB1 (known as EGFR), ERBB2, ERBB3, and ERBB4. Binding of ligands stabilizes the receptors in a dimeric form (comprising homo- and/or hetero-dimers) and facilitates the initiation of diverse downstream signaling events. This process is essential for the maintenance of the epithelium in both healthy, as well as inflammatory airway tissues. Repair and remodeling of damaged nasal epithelium is a highly organized and dynamic process. EGF is thought to be a key factor that drives the repair response within the epithelium [4]. In relation to that, EGFR can initiate a variety of signaling cascades when the epithelium is damaged or undergoing remodeling, by interacting with different ligands (e.g., EGF). EGFR can also crosstalk with other intracellular pathways in the presence of epithelial damage[6]. *In vitro* studies have demonstrated that EGF and EGFR promote wound closure in airway epithelial cells via both cell migration and proliferation [7–9]. In asthma, EGFR expression is increased in the epithelium and activation of EGFR contributes to airway epithelial repair [8, 10]. Polosa *et al*. demonstrated the localization of EGF and EGFR in the nasal epithelium of healthy inferior turbinates [11], while Burgel *et al*. demonstrated an up-regulation of EGFR in NPs, which was associated with goblet cell hyperplasia [12]. In addition, EGFR is believed to mediate neutrophil-dependent mucus (or mucin) production by goblet cells in both upper and lower airway mucosa [13, 14]. Unlike the other ERBB members, ERBB4 has the unique ability to undergo protein cleavage. Its intracellular domain (ICD) is able to translocate to the nucleus, thereby allowing it to participate in the regulation of gene transcription ([15, 16]. The functions of ERBB4 in lung epithelial cell development and alveolar surfactant production have been recently documented [17]. However, information regarding the ERBB4 expression in upper airways remains unclear.

NP tissues in Chinese patients exhibit a significantly higher proportion of neutrophilia than Caucasians [18], suggesting the presence of heterogeneity in the histological patterns of NP across different ethnic groups. We sought to extend the current knowledge regarding the role of EGF and EGFR by investigating the expression pattern of ERBB4 in NPs amongst Chinese patients. We also evaluated the relationship between these markers and epithelial change in NP, as well as their response to treatment with GC.

## Materials and Methods

### Patients and tissue samples

Fifty patients with NP were included in the study and the recruitment period of the patients was from Mar. 2012 to May. 2014. The diagnosis of NP was based upon the presence of relevant clinical symptoms, computed tomographic scans of the sinuses, as well as endoscopic findings. The patients were divided into two groups based on the following treatment arms: Forty patients (GC-naïve) had their GC therapy stopped 3 months prior to recruitment while another group of NP patients (n = 10) were treated with a course of oral prednisone (GC-treated; 10 mg three times per day for 10 days). Polyp biopsies were obtained during functional endoscopic sinus surgery. For the patients with GC treatment, polyp tissues were collected before and after the therapy, respectively. In the control group, 20 non-NP control patients with septal deviation who were free of sinus symptoms were recruited. Biopsies of inferior turbinate (IT) mucosa were obtained during septal surgery. None of the NP patients and control subjects had concurrent upper respiratory infections or other systemic diseases. The presence of atopy was evaluated by skin prick testing. All study subjects were recruited from the Department of Otolaryngology in the First Affiliated Hospital, Sun Yat-Sen University and the Department of Otolaryngology in the Qilu Hospital, Shandong University. Written consent forms were obtained from all participants and the procedure to conduct the study was approved by the Institutional Review Boards of the Sun Yet-Sen University, Shandong University, and National University of Singapore.

### mRNA expression in nasal tissues

A portion of NP or control nasal mucosa was stored in RNAlater solution (Applied Biosystems, Foster City, CA). Total cellular RNA was isolated from nasal tissues with RiboPure Kit (Applied Biosystems), followed by cDNA reverse transcription. mRNA levels of the selected genes were determined by TaqMan gene expression assays (Applied Biosystems): EGF (Hs00153181_m1), EGFR (Hs01076078_m1), ERBB4 (Hs00171783_m1), JUN (Hs00277190_s1). Relative gene expression was calculated using the comparative 2-ΔΔCt method [19], with GAPDH as a housekeeping gene.

### Histological, immunohistochemical, and immunofluorescent staining

Nasal tissues were fixed with formalin and embedded in paraffin. Samples were sectioned with microtome (Leica, Wetzlar, Germany). Slides were stained with hematoxylin and eosin (H&E) for general histology evaluation and eosinophil infiltration. Neutrophils were determined by staining with neutrophil elastase. The alcian blue (AB) was used to detect mucin levels in the tissue. Protein expression of EGF, EGFR, and ERBB4 was examined by immunohistochemical (IHC) staining, and co-localization of two proteins was observed by immunofluorescent (IF) staining. For both IHC and IF staining, slides were processed with Target Retrieval Buffer (Dako A/S, Glostrup, Denmark) followed by 10% serum blocking. Slides were stained with the following primary antibodies at 4°C overnight: mouse anti-human neutrophil elastase [NP-57] (DAKO A/S), mouse anti-human EGF monoclonal antibody [EGF-10] (Abcam, Cambridge, UK), rabbit anti-human EGFR monoclonal antibody [EP38Y] (Abcam), and mouse anti-human ERBB4 monoclonal antibody [HFR1] (Abcam). The slides were then incubated with DAKO EnVision+System-HRP (Dako A/S, Glostrup, Denmark) at room temperature for 30 min and developed by using Diaminobenzidine as substrate. For double IF staining, antibody of EGF or EGFR was combined with antibody of rabbit monoclonal against p63 (Abcam) or mouse monoclonal against p63, respectively; antibody of ERBB4 was combined with rabbit monoclonal against acetyl-α-tubulin (Cell signaling, Boston, MA), rabbit polyclonal against Foxj1 (Sigma-Aldrich, St. Louis, MO), and rabbit polyclonal against MUC5AC (Santa Cruz, Dallas, Texas), respectively. The sections were incubated with the set of primary antibodies and then incubated with Alexa Fluor 488 or 594 conjugated secondary antibodies (goat-anti mouse or rabbit IgG(H+L), molecular probes, Carlsbad, CA) in the dark at room temperature for one hour, and followed by mounting the slides with Antifade reagent with Dapi (molecular probes). Species- and subtype-matched antibodies were used as negative controls for both IHC and IF staining.

### Evaluation of nasal epithelium and inflammatory cell infiltration

In the evaluation of the epithelium structure, epithelium with more than 4 layers of cells was determined as epithelial hyperplasia [2]. The extent of mucus production by goblet cells, as well as the goblet cell number was evaluated by alcian blue (AB) staining. Goblet cell hyperplasia was defined as more than 2 layers of goblet cells in the epithelium. Eosinophils and neutrophils were randomly counted in three high power fields (HPF, 400 × magnification) from the lamina propria. Eosinophilia or neutrophilia was defined as eosinophils or neutrophils exceeding 10 respectively.

### Evaluation of protein expression levels in nasal epithelium

Three random fields of pseudostratified epithelium in sections from controls and 3 random fields of hyperplastic epithelium in sections from NP patients were chosen to evaluate the expression of each antigen under 400 × magnification using an optical microscope. Since there were different staining patterns among EGF, ERBB4 and EGFR in the epithelium, two types of the semi-quantitative scoring system were used to analyze the protein levels. For evaluation of EGF, the staining intensity graded as intermediate or high was defined as “normal” expression, while the intensity graded as negative or low was defined as “weak” expression. For evaluation of EGFR, a membrane staining was counted as positive, and for evaluation of ERBB4, a nuclear staining was counted as positive. The extent of immunoreactivity of EGFR and ERBB4 within the epithelium region was graded as Score 0 for negative staining; Score 1 for normal expression of 1–2 layers of positive cells; Score 2 for increased expression where there were more than 2 layers of positive cells. The observers who read the slides were blinded to the subjects’ clinical information.

### Statistical analysis

In GC-naïve NP vs. control groups (40 and 20), a standard difference of 1 with 80% power and 0.05/3 significant level can be detected; in GC-naïve vs. GC-treated NP groups (20 and 10), a standard difference of 1.4 with 80% power and 0.05/3 significant level can be detected; in GC-naïve and GC-treated NP groups (40 and 10), a standard difference of 1.3 with 80% power and 0.05/3 significant level can be detected. 0.05/3 = 0.016 was considered as significant level because of using Bonferroni adjustment for multiple comparisons. The data was analyzed using SPSS statistical software version 18.0 (SPSS Inc., Chicago, IL). The Mann-Whitney two-tailed test was performed to compare mRNA levels of EGF, EGFR, and ERBB4 among GC-naïve NP, GC-treated NP, and control groups in a pairwise comparison. Differences in categorical variables, including eosinophilia, neutrophilia as well asexpression levels of EGF, EGFR, and ERBB4 between the patient groups were compared by chi-square or Fisher’s exact test. Correlation analysis was performed using Spearman r. The *p* value < 0.05 was considered statistically significant.

## Results

The individual data points for the figures and table indicated in the Result sections were described in the S1 Table.

### Study population

The clinical characteristics and histopathological profiles of the NP g (both treated and untreated) and control group are summarized in Table 1. In the NP group, tissue eosinophilia and neutrophilia were found in 65% (26/40) and 58% (23/40) of the patients before GC treatment (GC-naïve or -GC) respectively. Epithelial remodelling is significant in GC-naïve NP patients—93% (37/40) of NP samples showed hyperplastic epithelium, 30% (12/40) had goblet cell hyperplasia, and 15% (6/40) exhibited squamous cell metaplasia. Amongst the 10 NP patients treated with GC (GC-treated or +GC), eosinophilia was present in 20% (2/10) and neutrophilia in 40% (4/10), a lower rate compared to the GC-naïve NPs. Epithelial remodelling was also not obvious in GC-treated NPs, where epithelial and goblet cell hyperplasia were present in only 30% (3/10) and 10% (1/10) of cases respectively.

**Table 1:** Patient characteristics and histopathological background.

	Healthy controls	NP patients (-GC)	NP patients (+GC)
Sample size (No.)	20	40	10
Age (y), median (1^st^-3^rd^ quartile)	31 (22–45)	46 (37–53)	40 (34–49)
Gender (M/F)	13/7	28/12	7/3
Atopy (No.)	1	5	1
Asthma (No.)	0	2	1
***Inflammatory cells***			
• Eosinophilic	0	26	2
• Neutrophilic	1	23	4
Epithelial remodeling*			
• Epithelial hyperplasia	2	37	3
• Goblet cell hyperplasia	1	12	1
• Squamous cell metaplasia	0	6	0

* Paraffin tissue sections from three control subjects were not evaluated due to the poor histology quality.

### Protein expression and localization of EGF, EGFR, and ERBB4 in nasal epithelium

**Fig 1:**
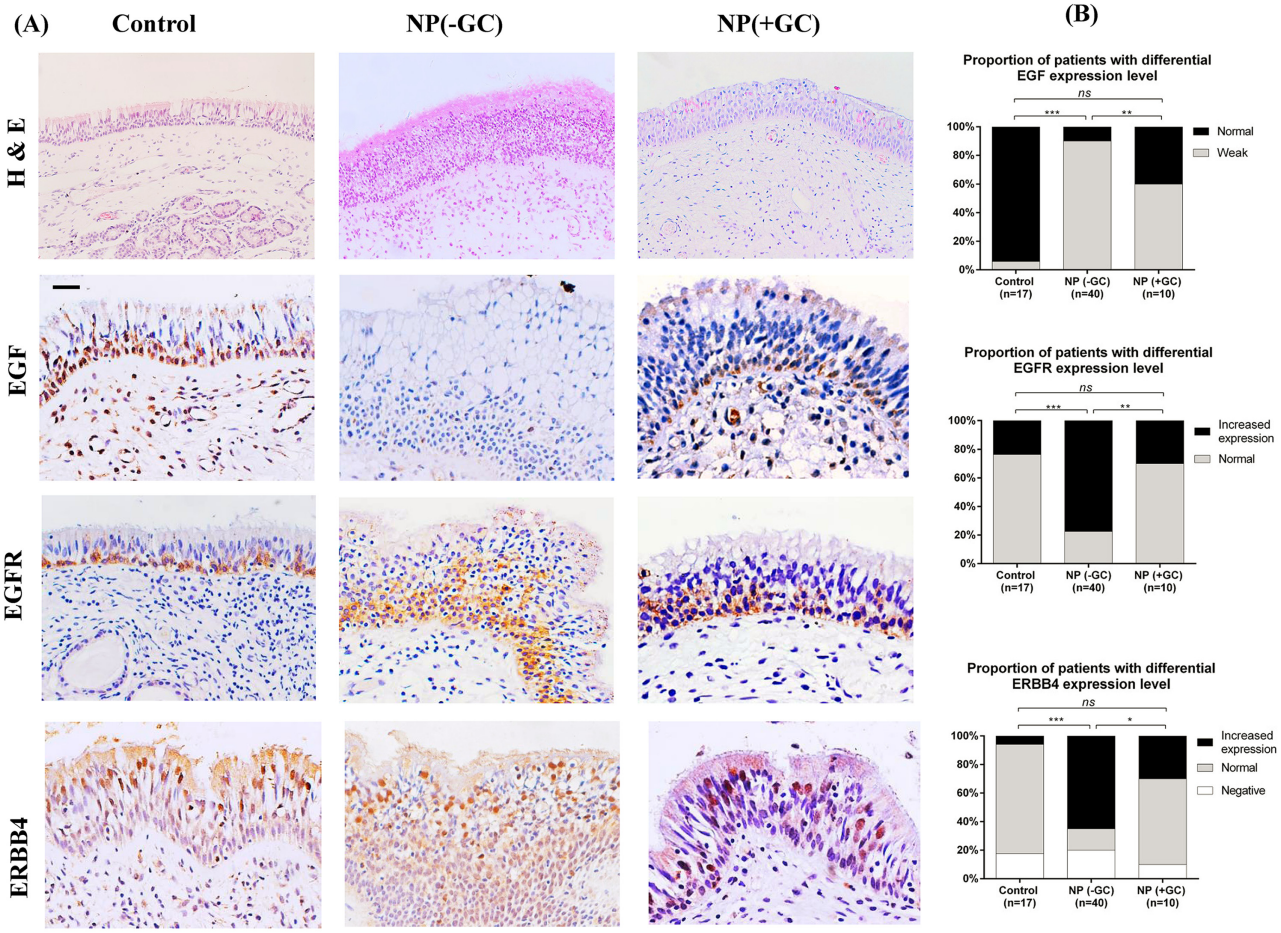
H&E staining (original magnification ×200) of the nasal mucosa from controls, GC-naïve NPs, and GC-treated NPs; expression and localization of EGF, EGFR, and ERBB4 in epithelium from each group (Panel A). Original magnification ×400 (Scale bar = 20 μm). Comparisons of the expression levels of these three markers were performed by chi-square or Fisher exact test (Panel B). *, *p* <0.05; **, *p* <0.01; ***, *p* <0.001; *ns*, no significance.

**Fig 2:**
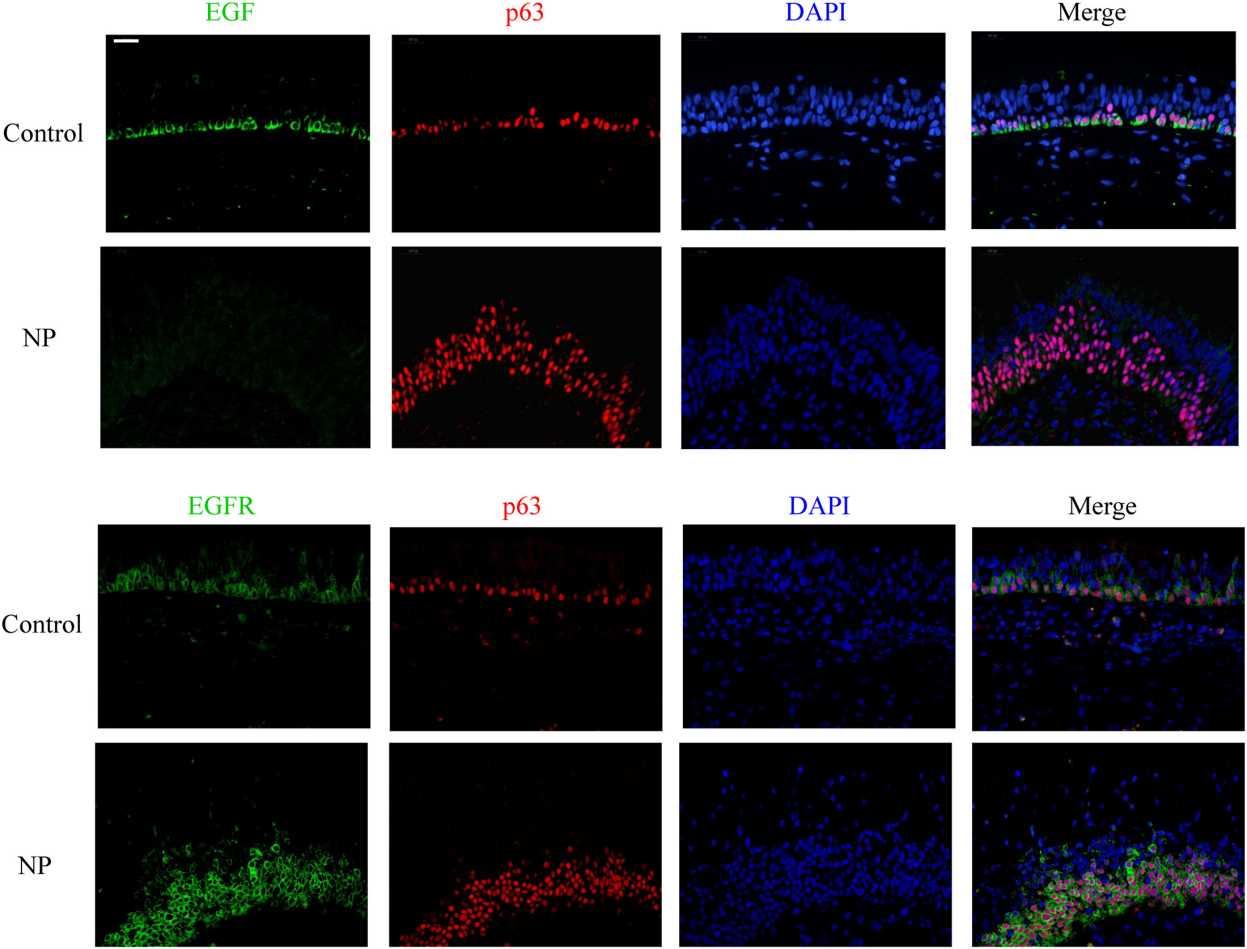
Double immunofluorescent staining of EGF (green) and EGFR (green) with p63 (red) in control and GC-naïve NP samples. DAPI (blue) stained in cell nucleus. Original magnification ×400 (Scale bar = 20 μm).

**Fig 3:**
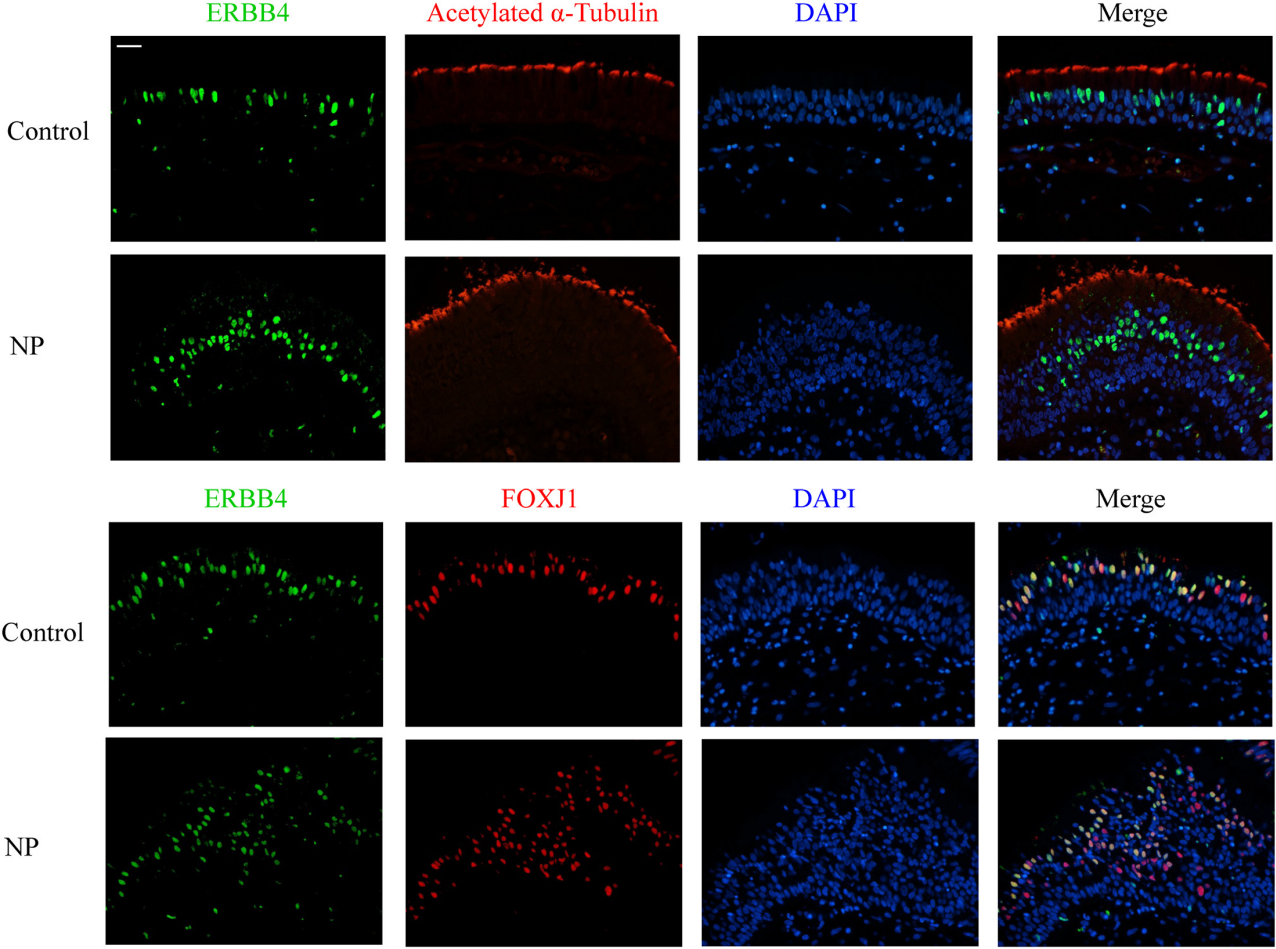
Double immunofluorescent staining of ERBB4 (green) with FOXJ1 (red) and Acetylated α-Tubulin (red) in control and GC-naïve NP samples. DAPI (blue) stained in cell nucleus. Original magnification ×400 (Scale bar = 20 μm).

Immunohistochemical analysis revealed a strong and consistent epithelial staining for EGF and EGFR in control subjects (Fig 1). EGF and EGFR showed a similar distribution within the nasal epithelium, mostly confined to the basal cell layer. Dual IF staining confirmed that EGF and EGFR were mainly seen in p63^+^ cells (Fig 2). The subcellular location of EGF and EGFR was restricted to the cell membrane (Figs 1 & 2). Interestingly, ERBB4 mainly localized within the nucleus of the columnar epithelial cells from the luminal surface, with only rare immunoreactivity present in basal cells (Fig 1). Therefore, the markers specific for ciliated cells were co-stained with ERBB4 antibody: Foxj1 localized mainly in the nucleus of the ciliated cells while acetylated α-tubulin marked the cilia structure. ERBB4 was frequently observed to localize within the ciliated cells based on the dual IF staining results (Fig 3).

The staining patterns of EGF, EGFR, and ERBB4 within the epithelium of NP tissues were different from those found within healthy nasal epithelium. The staining intensity of EGF was markedly decreased in both damaged epithelium and hyperplastic epithelium from NP mucosa; EGF was faint in the epithelium with basal cell hyperplasia (these cells stained positively for p63) (Fig 2). Weak expression of EGF was evident in 90% (36/40) of NP samples versus 5% (1/17) of controls, a marked difference (Fig 1). EGFR was co-localized with p63+ basal cells and its staining extensity was stronger in the NP epithelium with basal cell hyperplasia as compared to the healthy epithelium (Fig 2). Increased expression of EGFR was found in a significantly higher proportion of NP epithelium (78%, 31/40) as compared to controls (23%, 4/17) (Fig 1).

**Fig 4:**
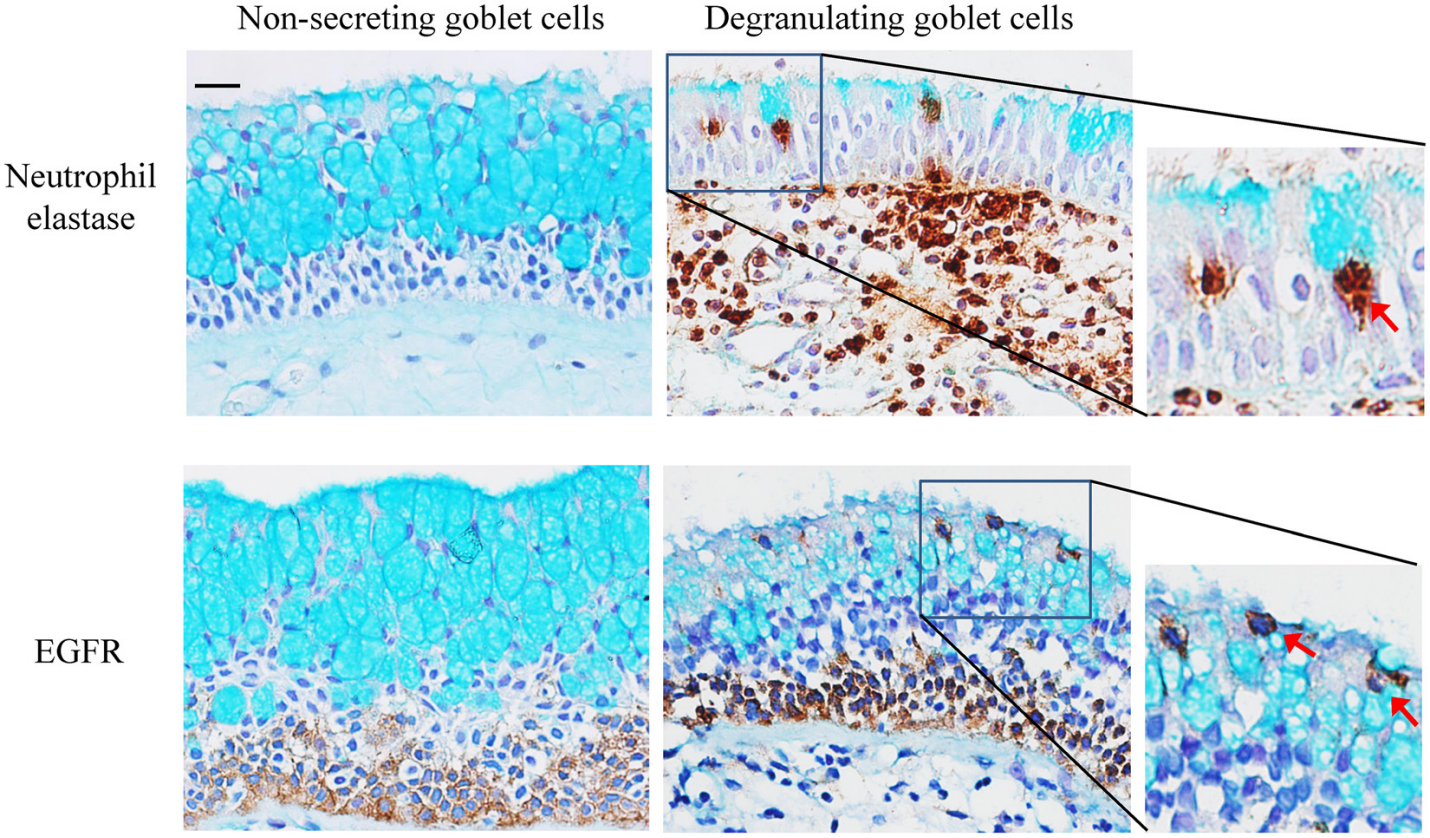
Neutrophil elastase or EGFR staining was counterstained by alcian blue. Red arrow indicated the degranulating goblet cells. Original magnification ×400 (Scale bar = 20 μm).

Recent literature has reported a possible hypothesis that goblet cell hyperplasia or hyperproduction was due to a neutrophil-elastase mediated activation of EGFR [20]. In order to examine the distribution of neutrophils, EGFR positive cells and goblet cells in nasal epithelium, all tissue sections were counterstained with alcian blue (AB). Interestingly, neutrophil recruitment was absent or rare in areas prominent with AB^+^ staining (i.e., non-secreting goblet cells), while neutrophil infiltration was high in the mild or negative AB^+^ stained area (some goblet cells undergoing degranulation) of the epithelium (Fig 4). With regards to the relationship between EGFR and goblet cells, the staining results showed that there was no co-localization of EGFR with non-secreting goblet cells, while a segment of goblet cells which were undergoing degranulation stained positively with EGFR (Fig 4).

In the hyperplastic epithelium from NP tissues, there was an increase of ERBB4 positive cells noted within the luminal area of the epithelium, and most of these cells expressed Foxj1 (Fig 3). It is interesting to note that Foxj1 as well as ERBB4 was not only expressed in surface cells positive for acetylated α-tubulin (i.e., ciliated cells), but were also found in cells within 3 layers of the apical layer of the remodeled epithelium (Figs 1 & 3). ERBB4 protein was not detected in 8 GC-naïve NPs, 3 controls, and 1 GC-treated NP–Increased expression of ERBB4 was found in 65% (26/40) of GC-naïve NPs versus 6% (1/17) of controls, and the difference was statistically significant (Fig 1).

After GC treatment, there was an improvement noted within the epithelial structure of most NP samples where a reduction of epithelial hyperplasia was demonstrated. (Fig 1)This was consistent with our previous findings [2]. A significant increase in the EGF expression level was detected in NP epithelium treated with GC, where 40% (4/10) of the patients showed strong EGF expression. This group of patients also exhibited a significant normalization in the expression levels of EGFR and ERBB4, where normal expression of EGFR and ERBB4 was found in 70% (7/10) and 60% (6/10) of GC-treated patients, respectively (Fig 1).

### Transcriptional levels of EGF, EGFR, and ERBB4 in nasal mucosa

**Fig 5:**
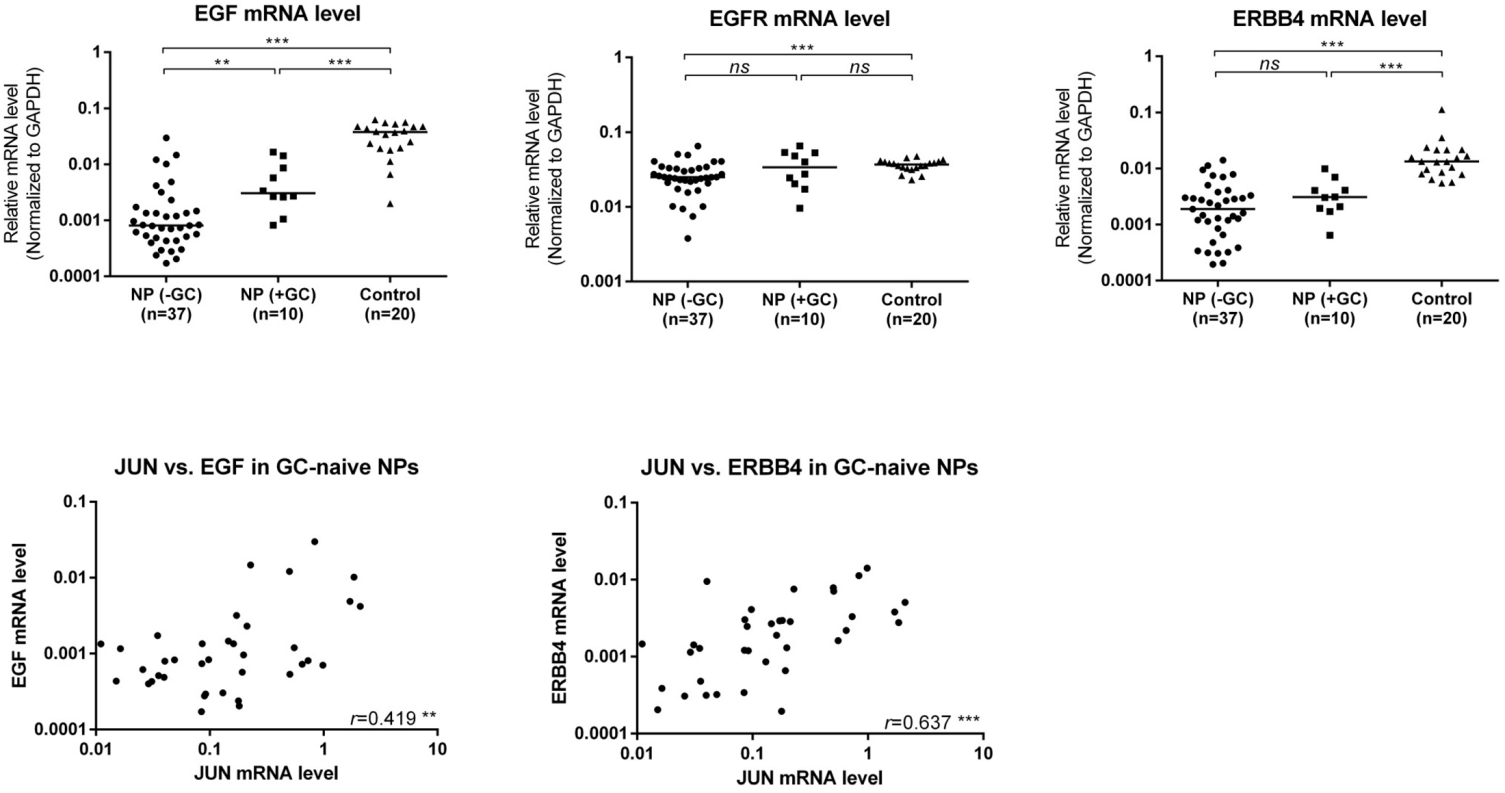
Quantitative determination of mRNA by means of real-time RT-PCR for EGF, EGFR, and ERBB4 in control, GC-naive and GC-treated patients with NPs. Solid lines show the median level of different groups. Statistical analysis for mRNA level comparison was performed with the Wilcoxon matched-pairs signed-rank test. Correlation between JUN and EGF or ERBB4 was evaluated by Spearman Rank analysis with correlation coefficient (*r*). *, *p* <0.05; **, *p* <0.01; ***, *p* <0.001; *ns*, no significance.

The mRNA levels of EGF (48.0-fold), EGFR (1.4-fold), and ERBB4 (7.1-fold) were significantly lower in GC-naïve NP than in controls (Fig 5). AP-1 transcription factor JUN was also tested and confirmed to be decreased in tissues from NP patients without GC treatment, which was in line with the previous results (Li CW et al., 2009). Correlation analysis showed JUN was positively correlated with EGF and ERBB4 in GC-naïve NPs, respectively (Fig 5). When compared to GC-naïve NPs, only EGF was significantly up-regulated at 3.7 fold in GC-treated NPs (Fig 5).

## Discussion

In the present study, we have demonstrated a differential expression pattern of EGF, EGFR, and ERBB4 in the nasal epithelium between patients with NP and control subjects. Basal epithelial cells are considered to play a critical role underlying the epithelial repair and remodeling process. The staining results showed a consistent localization of both EGF and EGFR in the basal cell layer of the healthy nasal epithelium. However, these two genes were differentially expressed in the damaged or remodeled epithelium from NP mucosa, where there was a down-regulation of EGF but an up-regulation of EGFR. Basal cells are considered the key driver for both epithelial repair and remodeling. Our previous published paper has determined a lower Ki67^+^ cell proportion among the p63^+^ basal cells in NP epithelium as compared to the healthy epithelium, indicating that the proliferation potential of NP epithelium is down-regulated [21]. Hence, the weak expression of EGF suggests that the growth of hyperplastic epithelium was abnormal, with dysregulation of the repair response. The increased expression of EGFR together with basal cell hyperplasia suggests that EGFR may play a role in mediating aberrant growth signaling in NP epithelium. Our results were in line with lower airway studies which revealed an inappropriate repair response in epithelium due to a lower EGF, but higher EGFR level [4, 10]. The varying expression patterns of EGF and EGFR in nasal airway epithelium during various inflammatory processes implies that their role in epithelial restitution and remodeling is diverse.

Another role of EGFR in airway epithelium is to regulate the mucin secretion from goblet cells, and this process is related to neutrophils which infiltrate the epithelium [13, 20, 22]. Our results are in agreement with these findings from previous studies. The degranulation was associated with a neutrophil infiltration within the epithelium. The expression pattern of EGFR within the apical layers of the epithelium was not consistent throughout the NP samples—there was an absence of EGFR in the mucosa with large areas of mucin-containing cells, but a presence of EGFR in areas of goblet cells undergoing degranulation. This phenomenon suggests that EGFR is involved in goblet cell degranulation within NP mucosa, but may not be directly involved in the process of mucin production within the cells. The two aspects of our EGFR results suggest that EGFR may not only play a role in differentiated cells (goblet cells) within nasal epithelium, but also have a role in the proliferation of basal epithelial cells.

One intriguing finding of the current study is the protein expression and location pattern of ERBB4 in nasal epithelium. *In vivo* and *in vitro* studies have indicated the important roles of ERBB4 ICD (locating in nuclear) in epithelial cell survival, proliferation, differentiation, and death [17, 23–25]. We are possibly the first group in observing the subcellular localization of ERBB4 in nuclei of ciliated cells within healthy nasal pseudostratified epithelium, indicating the role of this gene (supposedly the ICD epitope) in nasal epithelial cell differentiation. The increased expression of ERBB4 together with Foxj1 in the hyperplastic epithelium from NP mucosa suggests that ERBB4 may be implicated in the epithelial remodeling during chronic inflammation. The increase in ERBB4^+^/Foxj1^+^ cells in hyperplastic epithelium may represent the plasticity of Foxj1-expressing ciliated cells. Park et al. showed that Foxj1-positive cells participated in temporal squamous metaplasia and redifferentiation into mature columnar cells in response to epithelial damage in lung [26], while Turner et al. demonstrated a transdifferentiation of Foxj1-expressing progenitor cells into goblet cells by IL-13 treatment in bronchial airway epithelial cells [27]. Our recent study also reported an abnormal cilia architecture and ciliogenesis in hyperplastic nasal epithelium from NPs, which was associated with an increase in FOXJ1 expression [28]. Our results are in contrast to previous studies which showed no expression of ERBB4 mRNA in nasal epithelial cells from all tissue samples [11]. This difference could have resulted from different detection methods and materials. ERBB4 protein was also not detected in a certain portion of NP and control samples, which may be due to procedural inconsistencies in processing different batches of formaldehyde-fixed paraffin-embedded tissue. What remains to be investigated is whether or not there exists any interaction or crosstalk between ERBB4 and FOXJ1, and their functional roles in nasal epithelial cell differentiation, ciliogenesis and remodelling.

The current study reveals an increase in EGF, and a concomitant decrease in EGFR and ERBB4 expression in GC-treated NP epithelium. The close correlation between AP-1 (JUN) and two ERBB ligands (AREG and HBEGF), and their relationship to epithelial repair after GC-treated NPs has been described in our previous study [1]. EGF is not only the direct target gene of AP-1 (JUN), but also one of the major upstream signaling molecules to induce AP-1 translocation and activation via MAPK signaling pathways [29, 30]. The present results show a positive correlation between EGF and JUN. The lower expression levels of both JUN and EGF may reflect a decrease in the repair function within NP epithelium. This finding is consistent with prior *in vitro* studies which demonstrated a key role of the EGF/EGFR signaling activation pathway underlying the wound healing process in airway epithelium preceding GC treatment [9]. Hence, the increase in EGF following GC treatment in NP tissues may represent the enhancement in the epithelial repair process that GC promotes following injury of the nasal epithelium. Another beneficial effect of GCs is to modulate the epithelial remodeling in airway mucosa [2]. We previously found that GCs could reduce epithelial hyperplasia in NP mucosa as well as down-regulate the p63 (basal cell marker) and the TAp73 (differentiated/differentiating cell marker) genes [2]. The current study shows that EGFR and ERBB4 are mainly localized in basal and differentiated/differentiating cells, respectively. The return of EGFR and ERBB4 expression to normal levels following GC treatment suggests its effects on normalizing the hyperplastic epithelium in NPs. We also found in this study that there was a recovery of EGF expression, which together with the downregulation of EGFR and ERBB4 expression levels, resulted in the normalization of the nasal epithelium architecture.

There were, however, discordant results in protein and mRNA levels in EGFR and ERBB4. This could be due to different cellular sources for these genes in the tissue samples. However, the purpose of this study was to focus on the epithelial region and to compare the protein levels of the markers confined within the epithelium. One limitation for the current study is a relatively small number of the GC-treated NP patient group. Follow-up studies are needed in a large number of patients to verify the finding of these markers in patients treated with GCs.

**Fig 6:**
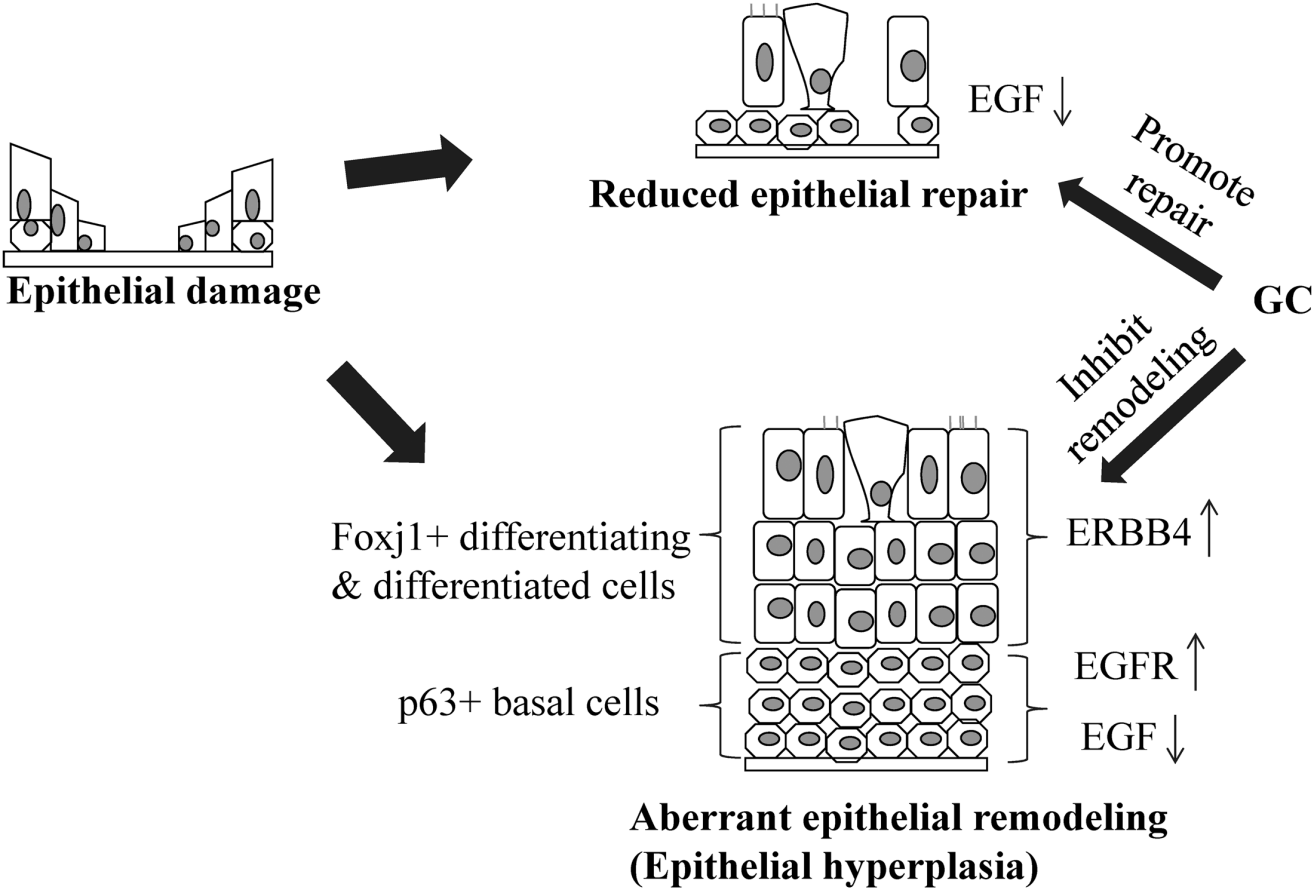
Schematic model of EGF, EGFR, and ERBB4 in epithelial repair and remodeling. Following the epithelial damage, a low level of EGF may lead to a reduced epithelial repair potential in NPs; while up-regulation of EGFR and ERBB4 may contribute to the epithelial hyperplasia. The potential effect of GC in NP epithelium is to restore the homeostasis of nasal epithelium.

To summarize the roles of EGF, EGFR, and ERBB4 in the repair and remodeling of nasal epithelial damage, we hypothesize the following (Fig 6):

1. As a result of epithelial damage in NP mucosa, theupregulation of EGFR expression represents a response to epithelial injury. As the EGF production by epithelial cells is insufficient, the disproportionate EGF to EGFR levels in the basal cells may lead to the reduction of epithelial repair and improper epithelial restitution (or aberrant epithelial remodeling);
2. Up-regulation of ERBB4 in the Foxj1+ cells suggests that it may have a role in another level of epithelial remodeling in differentiating or differentiated cells, other than basal cells;
3. The normalization of EGF, EGFR, and ERBB4 expression levels following GC treatment may represent its beneficial effects in restoring the homeostasis within NP epithelium.

In conclusion, the differential expression and localization patterns of EGF, EGFR, and ERBB4 within nasal epithelium may have a role in maintaining the homeostasis of healthy nasal epithelium. The low EGF activity and high levels of EGFR and ERBB4 in NP may contribute to the dysregulation in epithelial repair, and the process of abnormal epithelial remodeling. Also, the normalization of the function of these three markers may partly explain the beneficial effects of GCs on NP epithelium. Further *In vitro* cell models are required to elucidate the functional roles of these ERBB ligands/receptors, as well as their underlying signaling pathways involved in regulating the epithelial proliferation and differentiation of human nasal epithelium in normal, as well as in the diseased state.

## Supporting Information

S1 Table Full data points in the Result section. (XLSX)
